# Health-related quality of life among healthy elderly Iranians: a systematic review and meta-analysis of the literature

**DOI:** 10.1186/s12955-018-0845-7

**Published:** 2018-01-18

**Authors:** Sogand Tourani, Masoud Behzadifar, Mariano Martini, Aidin Aryankhesal, Masood Taheri Mirghaed, Morteza Salemi, Meysam Behzadifar, Nicola Luigi Bragazzi

**Affiliations:** 1 School of Health Management and Information Sciences, Iran University of Medical Sciences, Tehran, Iran; 2 Social Determinants of Health Research Center, Lorestan University of Medical Sciences, Khorramabad, Iran; 3 Section of History of Medicine and Ethics, Department of Health Sciences (DISSAL), University of Genoa, Genoa, Italy; 4 Health Management and Economics Research Center, Iran University of Medical Sciences, Tehran, Iran; 5 Department of Epidemiology, Faculty of Health & Nutrition, Lorestan University of Medical Sciences, Khorramabad, Iran; 6 School of Public Health, Department of Health Sciences (DISSAL), University of Genoa, Genoa, Italy

**Keywords:** Health-related quality of life, Systematic review and meta-analysis, Iran, Elderly

## Abstract

**Background:**

Health-related quality of life (HRQoL) measurement in elderly people can provide appropriate information for an optimal management of physical/mental conditions. The main objective of the present study was to quantitatively assess the HRQoL among healthy elder Iranian individuals as measured by the Short-Form 36 (SF-36) questionnaire, both overall and at the level of each its single component/domain.

**Methods:**

This study was designed as a systematic review and meta-analysis, following the "Preferred Reporting Results of Systematic Reviews and Meta-Analyses" (PRISMA) guidelines. Embase, PubMed/MEDLINE, ISI/Web of Science (WOS), Scopus, and Iranian databases such as MagIran, SID and Irandoc were mined from inception up to 1st September 2017. Also the grey literature (via Google Scholar) was mined. Two reviewers independently screened titles/abstracts, assessed full-text articles, extracted data, and appraised their quality using the "Strengthening the Reporting of Observational Studies in Epidemiology" (STROBE) checklist.

**Results:**

Twenty five studies were included. Mean overall HRQoL was 54.92 [95%CI 51.50–58.33], lower than the value found by studies done in other countries, especially in those economically developed. The sensitivity analysis indicated stability and reliability of results. Pooled scores of each HRQoL domain/sub-scale of the SF-36 questionnaire ranged from 49.77 (physical role functioning) to 63.02 (social role functioning).

**Conclusions:**

HRQoL among healthy elder Iranian individuals is generally low. Health policy-makers should put HRQoL among the elderly as a priority of their agenda, implementing ad hoc programs and providing social, economic and psychological support, as well as increasing the participation of old people in the community life and use their experiences.

**Electronic supplementary material:**

The online version of this article (10.1186/s12955-018-0845-7) contains supplementary material, which is available to authorized users.

## Background

Recent scientific achievements and medical advancements have resulted in increasing life expectancy and in ageing of the population, both in developed and developing countries [1]. This has led to a higher risk of developing chronic degenerative diseases. Iran is one of the developing countries, which, in the recent years, has seen a growing increase in the number of elderly together with declining fertility rates. In particular, the proportion of elderly population has significantly increased from 7.22% in 2006 to 8.20% in 2011, and, according to some estimates, is projected to further increase to 10.5% within 2025 and to 21.7% within 2050 [2].

Health outcome measurement and assessment enable to evaluate the performance of health plans and their impact, informing decision- and policy-makers in adopting scientific evidence-based, effective decisions [3]. Among the patient-reported outcomes (PROs), health-related quality of life (HRQoL) is the perceived quality of an individual’s health status and daily life, in terms of physical, mental and spiritual well-being. HRQoL represents a very useful indicator of overall health, capturing detailed information on both the physical and mental health status of subjects, and on their impact on quality of life. Various factors, such as gender and age as well as culturally prevailing values and standards, individual interests, social relationships, personal beliefs, economic and environmental features, can affect HRQoL [4, 5].

Ageing and ageing-related disease can impact too on both HRQoL and health-related costs. Due to limited financial resources in the health sector and the increased demand for healthcare services [6], HRQoL measurement in elderly people can provide both researchers and stakeholders with appropriate information for an optimal management of physical and mental conditions.

Extant studies conducted in different countries show that healthy ageing generally does not impact negatively on HRQoL, indicating that spending a long period in good quality of life is possible. Cultural differences do not usually influence the subjective dimension of quality of life, whereas they impact on its objective dimension [7].

Several Iranian studies have explored HRQoL in elderly population: however, they have produced contrasting findings. For instance, Tajvar and colleagues have found that HRQoL among elderly in Iran is particularly poor and low, whilst Tanjani and coworkers have concluded that HRQoL in Iran is well comparable with the values obtained in other countries [8]. To overcome the limitations that plague single primary studies (for example, in terms of small sample sizes), it is possible to carry out a systematic review and meta-analysis, which, pooling together different researches, increases their statistical power and enable to obtain more statistically robust and reliable findings.

As such, the present study was designed as a systematic review and meta-analysis of the literature and was conducted with the main objective of quantitatively assessing the HRQoL among healthy elderly Iranian individuals, both overall and of its single domain or component, since HRQoL is a multi-dimensional concept. The results of the present study could provide Iranian decision- and policy-makers with valuable insights for evidence-based decisions.

## Material and methods

The current systematic review and meta-analysis has been performed according to the “Preferred Reporting Items for Systematic Reviews and Meta-Analyses” (PRISMA) guidelines [9]. Two authors independently searched different scholarly databases: namely, Embase, PubMed/MEDLINE, ISI/Web of Science (WOS), Scopus, and Iranian databases such as MagIran, SID and Irandoc from 1st January 2000 up to 1st September 2017. Also the grey literature (via Google Scholar) was mined. Studies written in English or in Persian language were searched. Our search strategy was as follows: (“Quality of Life” OR “Health-Related Quality of Life” OR “Life Style” OR “QOL” OR “HRQoL”) AND (“Short-form questionnaire 36” OR “Questionnaire SF-36” OR “SF-36”) AND (“Elderly” OR “Aging”) AND “Iran”. Medical subject headings (MeSH) and wild-card options were used where appropriate. This search strategy was planned together with an information specialist.

In addition, reference lists of each identified study were examined for potentially eligible studies.

Inclusion criteria were: i) studies assessing HRQoL among health elderly people using the Short-Form 36 (SF-369 questionnaire, which is a validated, highly reliable and psychometrically sound instrument, comprising eight different domains/subscales: namely, physical functioning (PF), physical role functioning (PRF), bodily pain (BP), general health perceptions (GHP), vitality (VT), social role functioning (SRF), emotional role functioning (ERF), and mental health (MH) [6, 7]; and ii) studies reporting sufficient quantitative details such as standard deviation or standard error.

Exclusion criteria were: i) studies assessing HRQoL in sick elderly people; ii) studies with unclear results; iii) studies designed as clinical trials or reviews; iv) studies assessing overlapping populations (that is to say, dealing with the same populations); v) studies assessing HRQoL but not using the SF-36 questionnaire; and vi) studies not carried out in Iran.

Quality assessment of the included studies was evaluated using the 22-item “Strengthening the Reporting of Observational Studies in Epidemiology” (STROBE) checklist [10]. Studies were classified in good (score in the range 17–22), medium [8–16] and poor [1–7] quality studies.

Two authors independently extracted the following data from the selected studies: first author, publication year, sample size, mean age of the participants, mean overall HRQoL score and scores of each domain of the SF-36 questionnaire.

### Statistical analysis

The mean overall HRQoL score and the scores for each domain/sub-scale of the SF-36 questionnaire were estimated with their 95% confidence intervals (CI). To assess heterogeneity between studies I^2^ test was used [11]. If this amount was less than 50%, the fixed model was used, otherwise a stochastic model (IV-Heterogeneity) was used.

Since SF-36 is a multi-dimensional construct, with eight domains/sub-scales, which can show different aspects of HRQoL, scores for each component were collected and synthesized separately. Additionally, two summary measures, namely the Physical Component Summary (PCS) and the Mental Component Summary (MCS) scores were pooled together in order to obtain a direct picture of HRQoL.

A sensitivity analysis was performed to ensure the stability and robustness of the results [12]. To assess heterogeneity, meta-regression analyses were conducted on the basis of the sample and publication year. Egger’s test was used to investigate the presence of publication bias [13]. *P*-values < 0.05 were considered as statistically significant. All statistical analyses were performed using the software STATA (version 12.0).

## Results

**Fig. 1:**
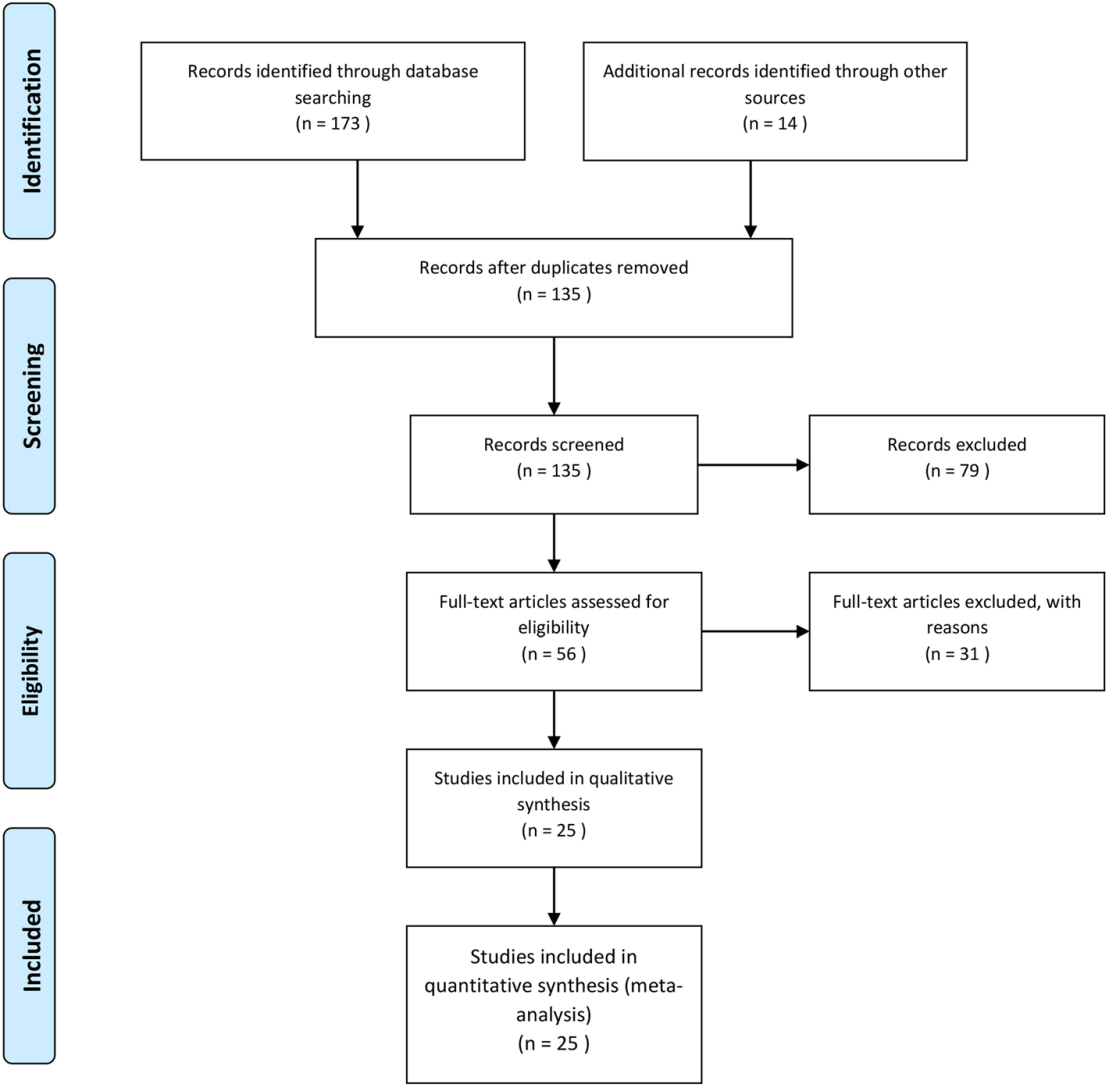
Flow-chart of the search strategy utilized in the current systematic review and meta-analysis

After the initial search and after deleting duplicates, 25 studies, meeting the inclusion criteria, were retained and analyzed [14–38], as shown in Fig. 1.

Among the included studies, 25 articles reported the overall HRQoL score, whilst 24 of them reported also the scores of each domain or sub-scale of the SF-36 questionnaire. The total number of participants in the current systematic review and meta-analysis was 12,328 elderly individuals. The sample size of the studies ranged from 56 to 5600 subjects. Characteristics of the included studies are shown in Table 1.

**Table 1:** Main characteristics of the studies included in the current systematic review and meta-analysis

Author	Year	City	Province	Sample size	Mean
Ghaderi	2014	Tabriz	East Azerbaijan	56	51.64
Farhadi	2011	Bushehr	Bushehr	69	32.1
Abdoli	2012	Tehran	Tehran	80	67.04
Shirvani	2016	Borujen	Chaharmahal and Bakhtiari	80	70.06
Naseh	2014	Shahrekord	Chaharmahal and Bakhtiari	87	41.56
Jadidi	2015	Tehran	Tehran	141	50.36
Abdollahi	2013	Sari	Mazandaran	153	70.39
Aghanuri	2012	Arak	Markazi	165	55.66
Mohammadiannia	2013	Bushehr	Bushehr	173	56.41
Heravi-Karimooi	2013	Tehran	Tehran	180	66.49
Salehi	2012	Tehran	Tehran	203	72.1
Heydari	2012	Sari	Mazandaran	220	46.031
Hedayati	2014	Shiraz	Fars	220	50.76
Hekmatpou	2014	Arak	Markazi	269	50.22
Zahmatkeshan	2012	Bushehr	Bushehr	360	47.75
Nejati	2008	Kashan	Isfahan	389	62.35
Vahdaninia	2005	Tehran	Tehran	396	53.9
Tajvar	2008	Tehran	Tehran	400	59.43
Darvishpoor Kakhki	2013	Tehran	Tehran	400	45.24
Salarilak	2013	Kamyaran	Kurdistan	400	60.62
Farzianpour	2016	Tehran	Tehran	400	49.7
Rakhshani	2014	Shiraz	Fars	500	50.8
Babak	2016	Isfahan	Isfahan	637	54.64
Hajian-Tilaki	2017	Babol	Mazandaran	750	56.8
Abbasimoghadam	2009	Tehran	Tehran	5600	51.589

Based on the STROBE checklist, 17, 5 and 3 studies were considered of high, medium and poor quality, respectively.

**Fig. 2:**
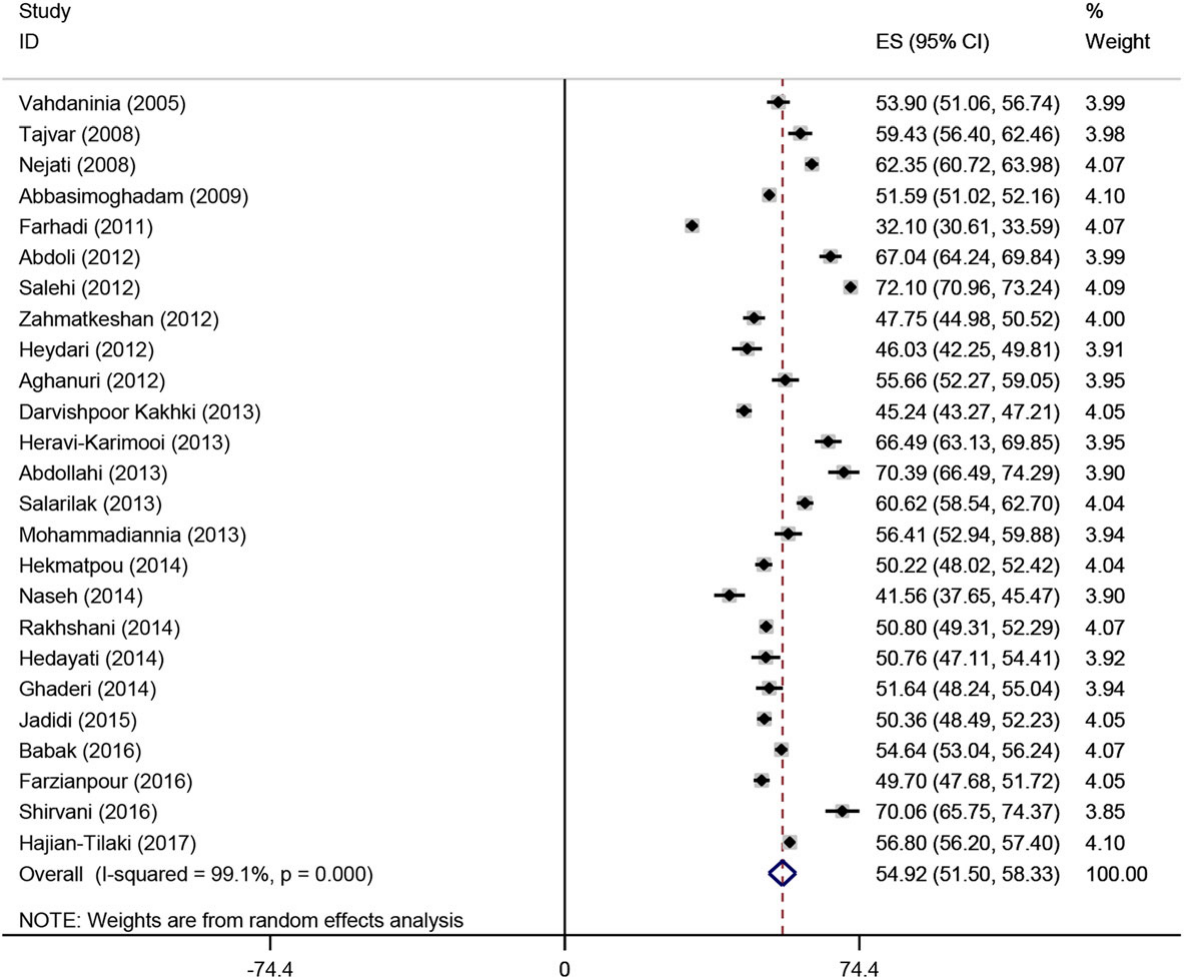
Forest plot of the studies included in the current systematic review and meta-analysis and reporting the overall health-related quality of life score assessed with the Short-Form 36 questionnaire

The pooled overall HRQoL score based on the random model was computed to be 54.92 [95%CI 51.50–58.33], with a statistically significant amount of heterogeneity (I^2^ = 99.1%) (Fig. 2).

**Fig. 3:**
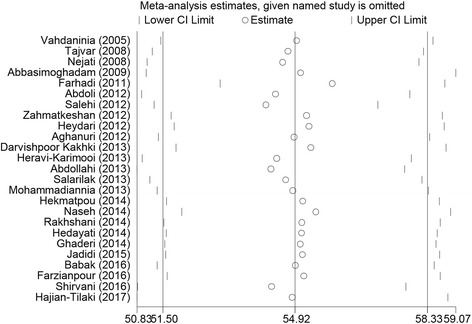
Sensitivity analysis of the studies included in the current systematic review and meta-analysis and reporting the overall health-related quality of life score assessed with the Short-Form 36 questionnaire

**Fig. 4:**
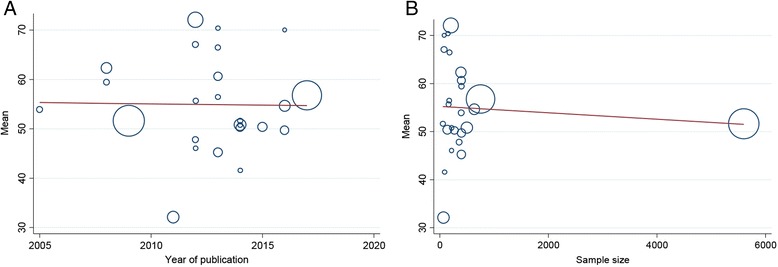
Meta-regressions carried out on the basis of publication year (**a**) and sample size (**b**)

The result of the sensitivity analysis is shown in Fig. 3, indicating stability and reliability of results. Findings of the meta-regression analyses stratified according to the year of publication and to the sample size are shown in Fig. 4a and b. Both meta-regressions were not statistically significant (*p*-value for publication year = 0.867, and *p*-value for sample size = 0.701).

**Fig. 5:**
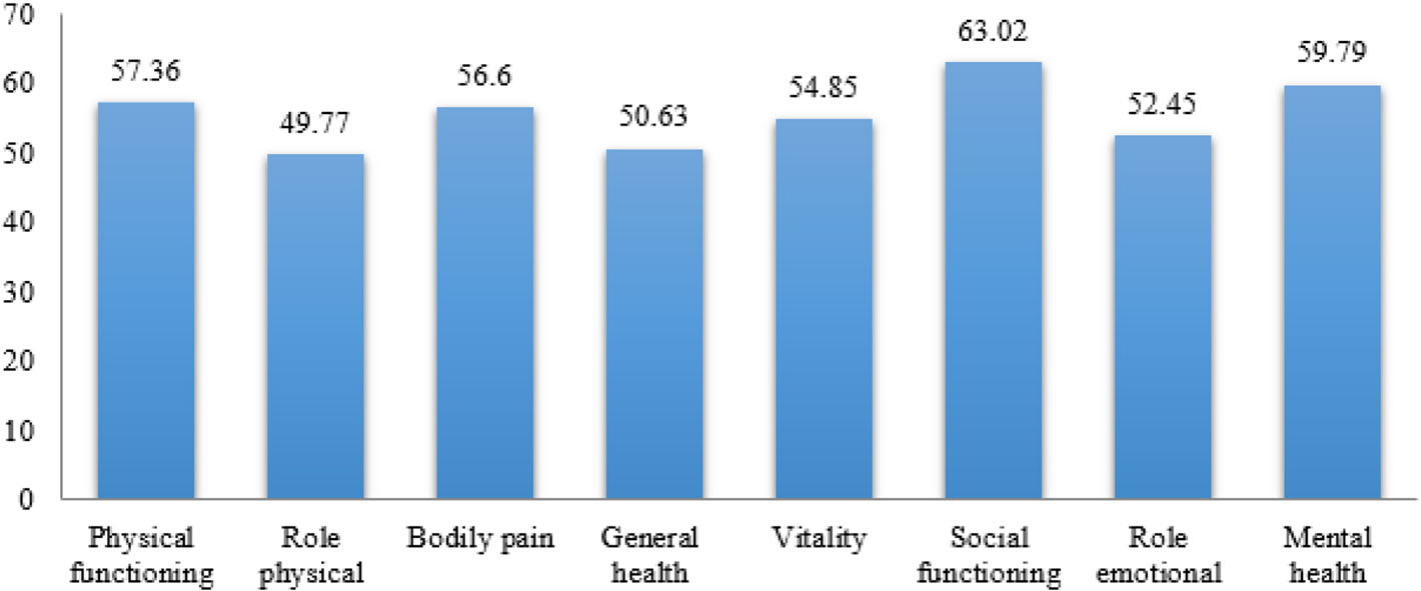
Pooled scores of each health-related quality of life domain/sub-scale assessed with the Short-Form 36 questionnaire

**Fig. 6:**
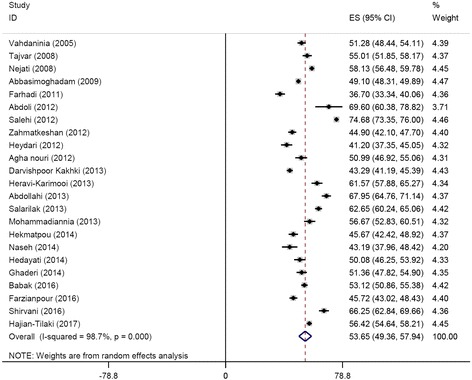
The Physical component summaries (PCS)

**Fig. 7:**
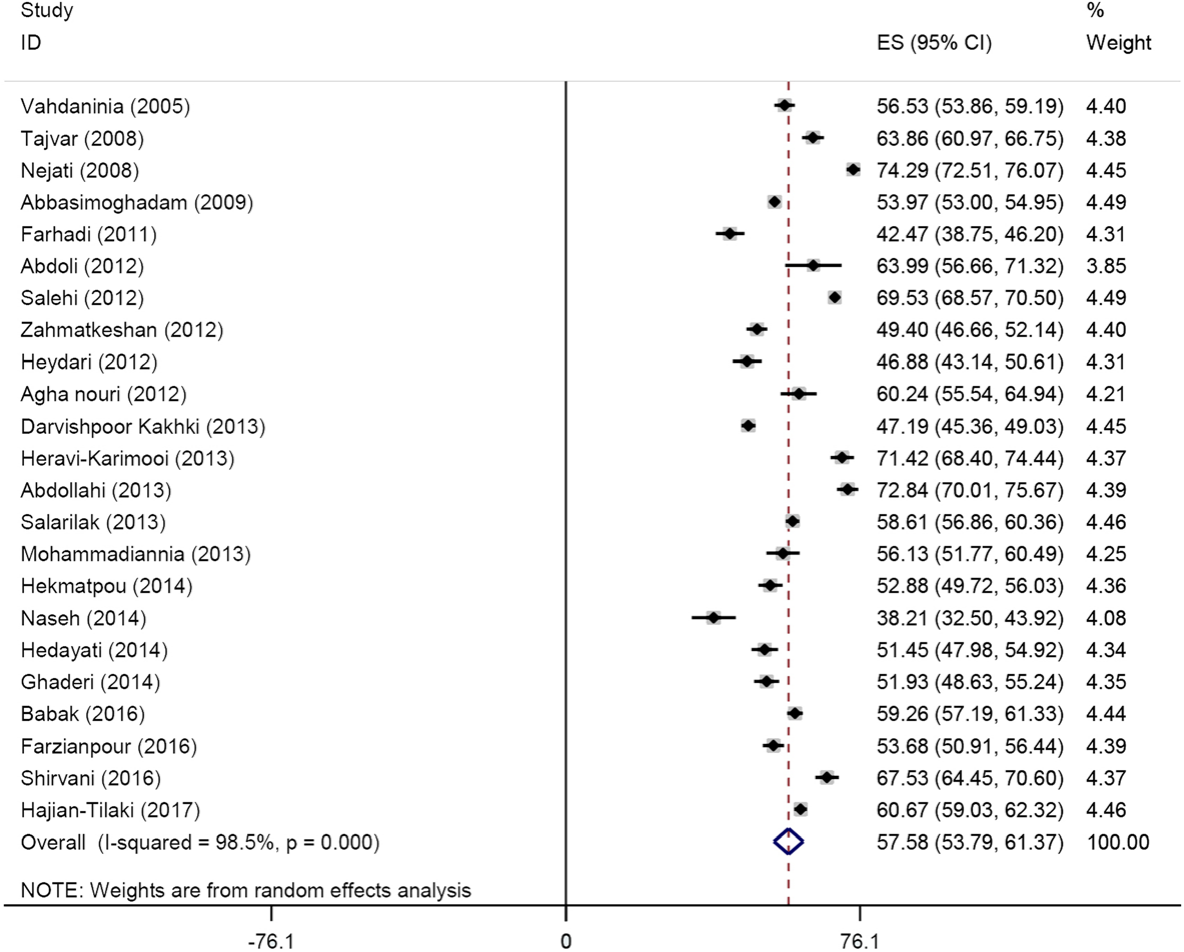
The mental component summaries (MCS)

Pooled scores of each HRQoL domain/sub-scale of the SF-36 questionnaire (ranging from 49.77 to 63.02) are shown in Fig. 5, while PCS (pooled ES 53.65 [95%CI 49.36–57.94]) and MCS (pooled ES 57.58 [95%CI 53.79–61.37]) scores are pictorially represented in Figs. 6 and 7, respectively.

Subgroup analyses were carried out stratifying the results according to the score of each domain/subscale of the SF-36 questionnaire (Table 2). Egger’s test value for the overall score and for the scores of the eight domains did not show any evidence of publication bias (overall *p* = 0.0948, PF *p* = 0.063, PRF *p* = 0.143, BP *p* = 0.690, GHP *p* = 0.529, VT *p* = 0.907, SRF *p* = 0.967, ERF *p* = 0.672, and MH *p* = 0.560).

**Table 2:** Subgroup analyses of the studies included in the current systematic review and meta-analysis and reporting the score of each health-related quality of life domain/sub-scale assessed with the Short-Form 36 questionnaire

Domains/subscales	Mean (95%CI)	I^2^	*P*-value
Physical functioning (PF)	57.36 (50.62 to 65.10)	99.6%	0.000
Physical role physical functioning (PRF)	49.77 (39.98 to 59.57)	99.7%	0.000
Bodily pain (BP)	56.60 (53.44 to 59.66)	97.7%	0.000
General health perceptions (GHP)	50.63 (47.75 to 53.50)	98.5%	0.000
Vitality (VT)	54.85 (51.72 to 57.98)	98.5%	0.000
Social role functioning (SRF)	63.02 (60.13 to 65.90)	97.8%	0.000
Emotional role functioning (ERF)	52.45 (42.86 to 62.04)	99.8%	0.000
Mental health (MH)	59.79 (55.60 to 63.98)	99.0%	0.000

## Discussion

The current study examined HRQoL in Iranian elderly people, using SF-36, a questionnaire that comprehensively assess various aspects of health [39].

Concerning mean overall HRQoL, in our study it was lower than the value found by, studies done in other countries, especially in those economically developed [40–46]. In Australia, data from the “Dynamic Analyses to Optimise Ageing” (DYNOPTA) project have shown that SF-36 scores range from 60.04 to 82.16, depending on the sub-scale. Similar results have been reported in the United Kingdom [47], in New Zealand [48], and in China [49], among others. In Germany, SF-36 scores ranged from 59.46 to 88.74 for males and from 57.25 to 84.24 for females, depending on the domain [50]. On the other hand, scholars in Chile have found rather low values (ranging from 49.1 to 55.7 for males and from 43.8 to 53.3 for females) [51].

Differences in health programs and in access to healthcare services provided can explain this discrepancy, as well as cultural, social, and economic factors, among others [52].

Concerning the different dimensions/sub-scales of the SF-36 questionnaire (Additional file 1), the findings of this study showed that GHP, PRF, and ERF reported the lowest scores [43, 53, 54], probably due to poor healthcare services for elderly people compared with the general population and lack of adequate funds [55], together with both individual and societal factors, since HRQoL is a multidimensional construct [56]. A low HRQoL among the elderly could be improved by targeted programs of health promotion, prevention and delivery of high-quality services.

Low HRQoL is associated with higher mortality rate. In elderly people, lack of movement increases the risk of suffering from cardiovascular disease, cancer, and diabetes, among others. A correct diet, regular exercise and periodic check-ups can maintain and promote an active and healthy life [57–59]. Reduced societal interactions and communications, as well as ageing-related psychological and behavioral features can explain a decreased score in the GHP domain/sub-scale [43]. Low physical health due to changes in lifestyle, economic status and lack of appropriate welfare services also contribute to a reduced HRQoL. Presence of partner during ageing could be of great help to individual happiness, preventing isolation, depression and premature death [60].

On the other hand, in our study, SRF and MH reported the highest scores. This could be attributed to the particular status of elderly people in the Iranian society, in that respect for the elderly is a religious and societal tenet [61].

Decision- and policy-makers should allocate resources in improving access to healthcare services and mental training among the elderly [62, 63], as Iran’s population has grown rapidly in the last years and is now significantly ageing.

In the last years, Iran has done many remarkable efforts in implementing various programs for health promotion, even though focusing less on the elderly [64]. It should be an onus to improve and enhance HRQoL among the elderly subjects. Iran, like many other developing countries, has limited financial resources in the health sector [65] and HRQoL assessment can play a major role in a rational resources allocation.

The present study had some limitations that should be properly cited, with the most important being the high heterogeneity between studies probably caused by differences in study conditions. Further, there is a dearth of data concerning HRQoL in some provinces of Iran and in rural environments, since most studies have been conducted in urban areas and in large cities.

On the other hand, the present study has some strengths, in that it adds to the extant literature, being, for example, more comprehensive and exhaustive than the systematic review and meta-analysis carried out by Farajzadeh and collaborators [66], which was based on 21 studies, where ours is based on 25 primary researches [67].

## Conclusion

The results of this study showed that HRQoL among healthy elderly Iranian individuals is generally low. Health policy-makers should put HRQoL among the elderly as a priority of their agenda, implementing ad hoc programs and providing social, economic and psychological support, as well as increasing the participation of old people in the community life and use their experience.

## Additional file

Additional file 1: Pooled scores of each health-related quality of life domain/sub-scale assessed with the Short-Form 36 questionnaire. (DOCX 99 kb)

## Abbreviations

BP: Bodily pain
CI: Confidence intervals
ERF: Emotional role functioning
GHP: General health perceptions
HRQoL: Health-related quality of life
MCS: Mental Component Summary
MeSH: Medical Subject Headings
MH: Mental health
PCS: Physical Component Summary
PF: Physical functioning
PRF: Physical role functioning
PRISMA: Preferred Reporting Items for Systematic Reviews and Meta-Analyses
PROs: Patient-reported outcomes
SF-36: The Short-Form 36
SID: Scientific Information Database
SRF: Social role functioning
STROBE: STrengthening the Reporting of OBservational studies in Epidemiology
VT: Vitality
WOS: Web of Science
