# Supplementary material for: Health-related quality of life among healthy elderly Iranians: a systematic review and meta-analysis of the literature
Source: Health Qual Life Outcomes. 2018 Jan 18;16:18. doi: 10.1186/s12955-018-0845-7 (PMC5774099; doi:10.1186/s12955-018-0845-7)
Supplement: Additional file 1: — Pooled scores of each health-related quality of life domain/sub-scale assessed with the Short-Form 36 questionnaire. (DOCX 99 kb) [file 12955_2018_845_MOESM1_ESM.docx]

**Pooled scores of** **each health-related quality of life domain/sub-scale assessed with the Short-Form 36 questionnaire**
